# Climate shocks and nutrition: The role of food security policies and programs in enhancing maternal and neonatal survival in Niger

**DOI:** 10.1111/mcn.13566

**Published:** 2023-10-04

**Authors:** Shelley Walton, Nasreen S. Jessani, Heather Jue‐Wong, Elizabeth A. Hazel, Nadia Akseer, Almamy Malick Kante, Ousseini Youssoufa, Rebecca Heidkamp, Assanatou Bamogo, Agbessi Amouzou

**Affiliations:** ^1^ Johns Hopkins Bloomberg School of Public Health, International Health Department Johns Hopkins University Baltimore Maryland USA; ^2^ Centre for Evidence Based Health Care Stellenbosch University Cape Town South Africa; ^3^ Institut National de la Statistique du Niger (INS‐Niger) Niamey Niger

**Keywords:** climate change, food security, maternal, neonatal, Niger, nutrition, resilience, subnational

## Abstract

Niger is afflicted with high rates of poverty, high fertility rates, frequent environmental crises, and climate change. Recurrent droughts and floods have led to chronic food insecurity linked to poor maternal and neonatal nutrition outcomes in vulnerable regions. We analyzed maternal and neonatal nutrition trends and subnational variability between 2000 and 2021 with a focus on the implementation of policies and programs surrounding two acute climate shocks in 2005 and 2010. We used four sources of data: (a) national household surveys for maternal and newborn nutritional indicators allowing computation of trends and differences at national and regional levels; (b) document review of food security reports; (c) 30 key informant interviews and; (d) one focus group discussion. Many food security policies and nutrition programs were enacted from 2000 to 2020. Gains in maternal and neonatal nutrition indicators were more significant in targeted vulnerable regions of Maradi, Zinder, Tahoua and Tillabéri, from 2006 to 2021. However, poor access to financial resources for policy execution and suboptimal implementation of plans have hindered progress. In response to the chronic climate crisis over the last 20 years, the Nigerien government and program implementers have demonstrated their commitment to reducing food insecurity and enhancing resilience to climate shocks by adopting a deliberate multisectoral effort. However, there is more that can be achieved with a continued focus on vulnerable regions to build resilience, targeting high risk populations, and investing in infrastructure to improve health systems, food systems, agriculture systems, education systems, and social protection.

## Key messages

- Niger is ranked as the most vulnerable country in the world for exposure and sensitivity to the negative effects of disasters and climate change. Acute environmental shocks in 2005 and 2010 elevated government and development partner responses to the ‘food security crises’.
- Multisectoral policy and program investments in vulnerable regions played a meaningful role in improving food security and subsequently maternal and neonatal nutrition but progress is slow or stagnant.
- The Nigerien government, program implementers, and donors should prioritize investments in infrastructure and shock resilience to improve health systems, food systems, agriculture systems, education systems, and social protection, particularly in high risk populations.

## Graphical abstract

Multisectoral nutrition and food security policy and program investments in response to climate shocks in vulnerable regions have played a meaningful role in improving food security and maternal and fetal growth in Niger over the last 20 years.

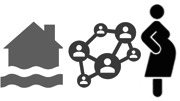

## 1 INTRODUCTION

### 1.1 Maternal and neonatal mortality and nutrition

Historically, Niger has been afflicted by high poverty rates, environmental crises, food insecurity, high fertility and political instability. Yet, World Health Organization (WHO) estimates show that the maternal mortality ratio (MMR) in Niger has decreased by 37% over a decade and a half, from 813 maternal deaths per 100,000 live births in 2000 to 509 per 100,000 in 2017 (WHO et al., 2019). The neonatal mortality rate (NMR) has also shown a rapid and steady decline from 43.1 deaths per 1000 live births in 2000, to 24.3 in 2019 (The World Bank, 2022). Niger outranks other West African countries in the reduction of neonatal and maternal mortality over this period, even those with similar or better economic progress (Countdown to 2030, 2021).

This research is part of a wider Exemplar in Maternal and Neonatal Mortality study across seven countries to understand factors at the individual, household, community and national levels that were key to reducing neonatal and maternal mortality over the last 20 years (Exemplars in Global Health, 2022).

Maternal and neonatal mortality is affected by nutritional status and practices. Maternal anaemia, short stature, low body mass index and poor maternal weight gain can lead to poor birth outcomes and increased mortality (Barker et al., 1993; Rahman et al., 2016). Early initiation of breastfeeding, within 1 h of birth, protects the newborn from acquiring infection and reduces newborn mortality and exclusive breastfeeding reduces infectious disease, diarrhoea, respiratory infections, meningitis and neonatal sepsis (Debes et al., 2013). Further, low birthweight babies account for 60%–80% of all neonatal deaths (Katz et al., 2013).

As mortality rates have fallen dramatically in Niger, the national rates of child stunting, child wasting, and anaemia in pregnant women have made slower progress. The national stunting prevalence was 52.1% in 2000 and 46.7% in 2020, wasting was 16.2% in 2000 and 9.8% in 2019, and anaemia during pregnancy was 62% in 2006 and 59% in 2012. These rates reflect some progress on the national level but mask the situation at the subnational level.

### 1.2 Environmental crises and food security contributions to malnutrition

Most Nigerien's livelihoods depend on agriculture, and frequent droughts in Niger have led to chronic food insecurity resulting in high malnutrition rate (USAID, 2014). Approximately 2.5% of the rural population in Niger is severely food insecure, roughly 13.2% of individuals are moderately food insecure, and 33.3% are classified as ‘at risk’ for food insecurity (Institut National de la Statisque [INS] and Systemes d'Alerte Pre‐coce [SAP], 2015). The 2019 Global Hunger Index ranked Niger 101 out of 117 countries despite improvements in overall score from 52.1 (extremely alarming level of hunger) in 2000 to 30.2 (serious level of hunger) in 2019.

Some factors contributing to food insecurity in Niger include high fertility, poor agricultural yield and quality, and climate change (USAID, 2014). For example, Niger has the highest birth rate in the world, about 7.8 children per woman, contributing to the pressure on food systems and food security (USAID, 2014). Currently, 80% of livelihoods in Niger are driven by agriculture and livestock (The World Bank, 2021). Yet, Niger has only one harvest per year and an annual lean period from June to October (The World Bank, 2021). The Notre Dame Global Adaptation Initiative (ND‐GAIN) ranks Niger as the most vulnerable country (182/182) in the world for exposure and sensitivity to the negative effects of disasters and climate change (ND‐Gain, 2023). Climate change and vulnerability changes are known to negatively impact nutrition and mortality through reduced crop production and poor dietary diversity and nutrient quality.

While there has been a longstanding need for food aid in Niger, there were two acute and severe climate shocks since 2000 that affected population vulnerability to malnutrition and mortality. In 2005 a short rainy season, locust infestations, and crop failure contributed to severe food insecurity and food aid requirements at levels 20 times higher than before (Global Humanitarian Assistance, 2022). An estimated 2.5 million (18.5%) Nigeriens were affected by the food crisis including more than 261,000 pregnant and lactating women, 32,000 children were severely malnourished and 160,000 were moderately malnourished (USAID, 2006). Similarly, in 2010, the year a new government came into power in Niger, the World Food Programme (WFP) proclaimed the worst hunger crisis in Niger's history with almost half of the population in desperate need of food (BBC, 2010). WFP also reported that 20% of children were acutely malnourished, which is above the 15% threshold for declaring an emergency.

### 1.3 Food insecurity and nutrition policies and programs linked to maternal and neonatal mortality

In line with the broader Exemplars in Maternal and Neonatal Mortality study, our analysis aims to identify the role of country‐specific policies and programs across time and administrative levels on the structural, household, and individual level, as it relates to food security, nutrition, and maternal and neonatal mortality. Understanding these linkages provides the pathways of impact that policies and programs target at three different levels. Structural level environmental conditions—such chronic climate shocks, through droughts and floods, subpar soil quality, insufficient pasture for animals, price spikes, and political instability—impact household level food available by impacting production and incomes as well as food prices and increase needs for food assistance. These link to individual level changes—food consumption and health status—and ultimately, when policy and humanitarian responses are insufficient, impact individual maternal and neonatal nutrition, health, and survival.

This study analyzes maternal and neonatal nutrition trends and subnational variability from the early 2000s with a special focus on the implementation of policies and programs surrounding the two acute crises in 2005 and 2010.

## 2 METHODS

### 2.1 Data sources

We used data from national household surveys that measure coverage of maternal and newborn nutritional indicators: two Demographic and Health Surveys (DHS 2006—Institut National de la Statistique [INS] & Macro International Inc, 2007, and 2012—INS & ICF International, 2013), the National Fertility and Under 5 Mortality Survey (ENAFEME 2021; Institu National de la Statisque, 2021) and 11 Standardized Monitoring and Assessment of Relief and Transitions (SMART from 2006 to 2020 [INS & Ministere de la Sante Publique, 2020]).

We conducted a document review of food security and climate shock reports in English and French and pulled relevant national level data; subnational food security data were limited. Documents included grey literature from implementing partners and donors, government policy documents and reports, and scientific papers on nutrition interventions in Niger.

Thirty key informant interviews and one focus group discussion were conducted as part of the Exemplars in Maternal and Neonatal Mortality study and reported elsewhere (Kante et al., in press).

### 2.2 Analysis

We analyzed the following nutrition indicators for women (body mass index [BMI], anaemia during pregnancy, and iron folate supplementation coverage) and neonates (early initiation of breastfeeding, exclusive breastfeeding, breastfeeding practices, and used underweight, stunting, and wasting trajectories from birth up to age 5 years as a proxy to demonstrate undernutrition and growth faltering patterns in the first 5 years of life). For each indicator, national and subnational trends and differences were computed. The intervention coverage data and selected nutritional status data from DHS were reanalyzed by the International Center for Equity in Health (ICEH, Pelotas, 2021) for consistent indicator definition and subnational regions over the evaluation period.

To understand the growth faltering from birth to up to 5 years of age, Victora et al. (2010) described the child age versus HAZ (or WHZ, WAZ) curves for all regions of the world using national household surveys. We applied this approach in Niger to explore birth disadvantage subnationally.

Our definitions of food insecurity are based on the FAO Food Insecurity Experience Scale which is an experience‐based metric of food insecurity severity (Cafiero et al., 2018).

Niger has very limited data on nutritional maternal and newborn indicators before 2006. The DHS 1998 included only early initiation of breastfeeding and women's weight indicators. Statistical analysis focused on 2006–2021 because of data availability.

The document review included the evolution of food security and nutrition policy investments and program implementation in Niger between 2000 and 2020. We focused on national and subnational programs and policies related to nutrition and food security and their potential impacts on maternal, newborn, and child health. We also reviewed documents related to reporting on climate shocks (e.g., floods, droughts, infestations) in Niger between 2000 and 2020. We classified subnational climate shocks, duration, and intensity.

For the qualitative component, the interview transcripts were coded and organized by major themes outlined in the interview guide. Nutrition was not initially determined as the main focus of the Exemplars in Maternal and Neonatal Mortality study (Kante et al., in press). However, nutrition was raised as an important topic when key informants were asked about health and nonhealth factors related to maternal and neonatal mortality reductions in Niger. Quotes specific to the nutrition story were extracted from the transcripts, deidentified, and included in the analysis and paper where relevant.

### 2.3 Ethical clearance

The quantitative analyses used publicly available data with no ethical clearance required. Ethical clearance for the qualitative key informant interviews was obtained from the Niger ethical review board of the Ministry of Health and was exempt by the Johns Hopkins Bloomberg School of Public Health's institutional review board.

## 3 RESULTS

### 3.1 Food security and nutrition policy investments (2000–2020)

**Figure 1:**
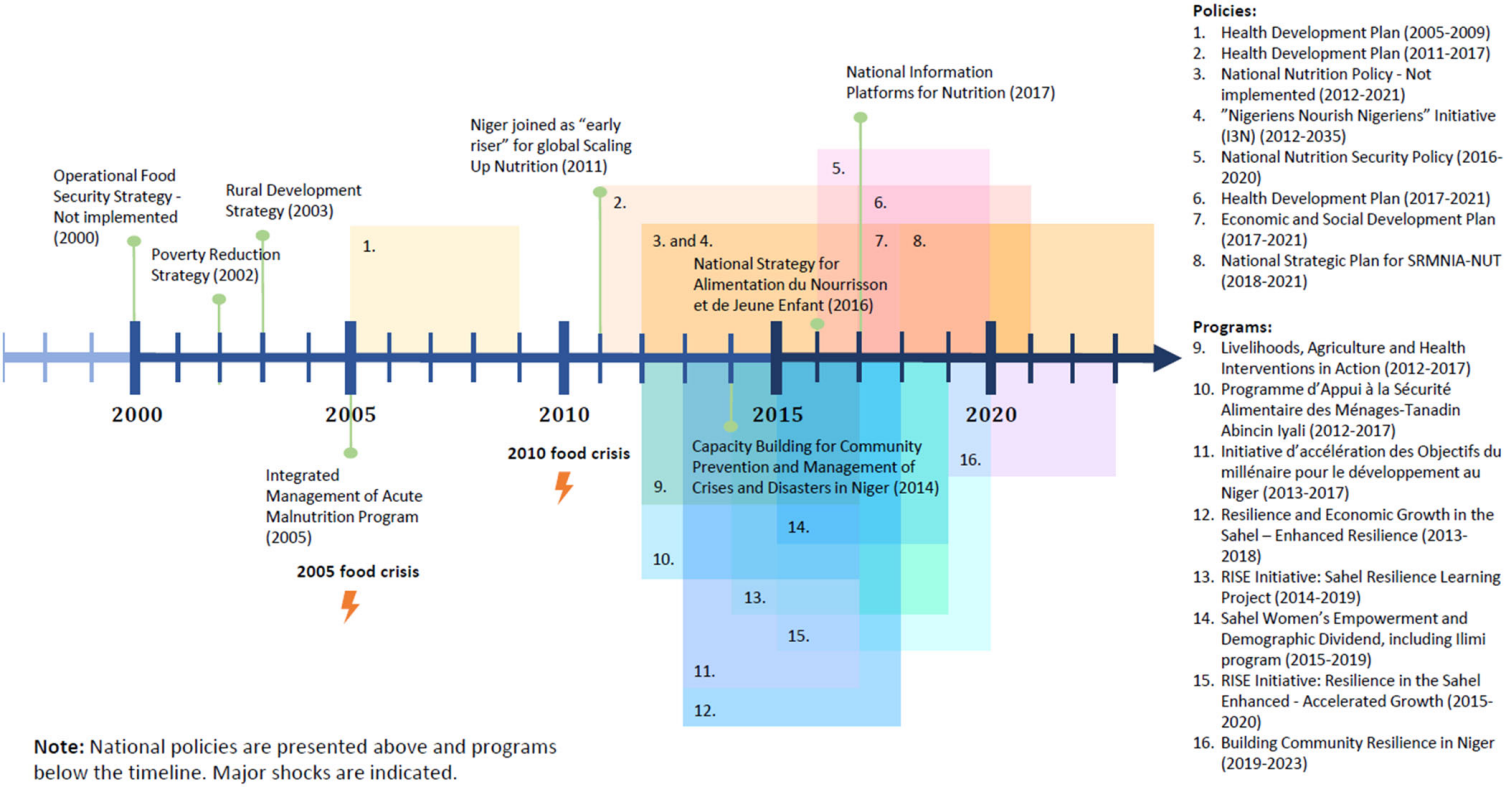
Policies and programs related to nutrition and food security in Niger, 2000–2020.

Since 2000, and really gaining momentum in 2010, the Nigerien government, with local and international development partners, has ensured a concerted effort to address acute and chronic malnutrition. The advent of a new government in 2010 raised the profile of nutrition leading to a plethora of policies and initiatives (Figure 1). Government‐led policy changes included the institution of a High Authority for Food Security (HASA) in 2012, the president and government's 3N initiative ‘Nigeriens Nourish Nigeriens’ launched in 2012, and the inclusion of Niger in the Scaling Up Nutrition (SUN) Movement starting in 2011. Structural governance adaptations, such as raising the status of the Nutrition Division to a directorate within the Ministry of Health, were matched with integrated national policies and focused programs at the subnational level.

Policy development efforts were often supported and complemented by international organizations, as noted by this respondent (and summarized in Supporting Information: Table 1):

“The package of activities covers 37 municipalities in the WFP convergence zone, and for the past 15 years the WFP has been assisting pregnant women. Rates of malnutrition … the death rate, and the child's small weight of life in the first month (have decreased).” WFP interviewee

One EU supported international initiative, National Information Platforms for Nutrition, leveraged the 3N initiative, SUN, and data analysis and coordination of the Institut National de la Statistique in Niger to support data‐informed policies and investments for nutrition (NiPN, 2023).

Significant increases in investments for nutrition, food security, and household and community resilience were spurred by the 2010 food crisis which drew international attention to the dire situation in Niger. These efforts have been greatly appreciated by the government as noted here:

“I see the effort of WFP and UNICEF, in Niger if there is not this support many children will die, so nutrition program there whether women or children, a program that will focus on nutrition and therefore will intervene on food security.” Technical Officer, Niger Ministry of Public Health, Population and Social Affairs

### 3.2 Food security and nutrition program implementation (2000–2020)

Over the last 20 years maternal, newborn, and child nutrition, food security, and resilience programs in Niger were at the national level (three programs) or in targeted agropastoral regions with high burdens of food insecurity and malnutrition including Maradi (six programs), Zinder (six programs), Tillaberi (three programs), and Tahoua (one program) (Table 1). Several programs intentionally targeted high disaster risk regions to improve health and nutrition indicators and build the resilience of households and communities to withstand shocks.

**Table 1:** Subregional summary of improvements in maternal and newborn nutrition and food security indicators from 2006 to 2021.

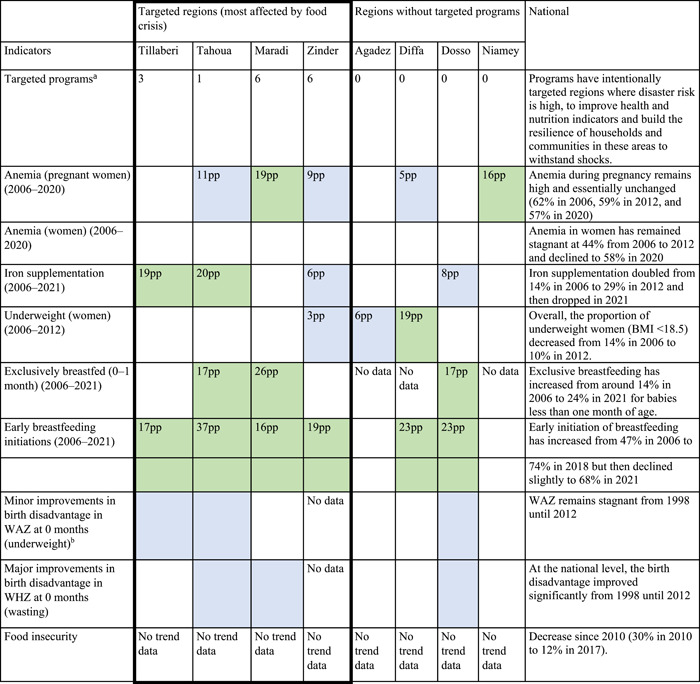

*Note*: Green box = significant improvements in subregions relative to national trend; Blue box = moderate improvements in subregions relative to national trend.

Abbreviation: pp, percentage points and values in cells depict the pp improvement.

^a^Number of targeted programs focused on maternal, newborn, and child nutrition, food security and resilience programs in Niger.

^b^Minor improvements in birth disadvantage and faltering in the first 1 year of life in WAZ (underweight).

Food security policies and programs also targeted regions that were most impacted by food crises and insecurity, such as Maradi, Zinder, Tillaberi, and Tahoua. However, poor access to financial resources remains a barrier and the execution and implementation of plans have sometimes been suboptimal. As one respondent noted:

“We are champions of planning, we are champions of budgeting and everything that follows, but what we lack here is perhaps a delay in implementation…in Niger it's not the policies that we lack.” Technical Officer, Division of Maternal Health, Department of Maternal and Child Health

Such programs reaching multiple agropastoral regions include the Resilience and Economic Growth in the Sahel—Enhanced Resilience (RISE‐REGIS‐ER) which operated, across Maradi, Tillaberi and Zinder from 2013 to 2018 and reached 97,945 children under five with nutrition programs, 19,123 children under two with community level nutrition interventions, 14,175 pregnant women with nutrition‐specific interventions, and trained 177,407 people in child health and nutrition. This was complemented by the RISE Initiative: Sahel Resilience Learning Project (SAREL) (2014–2019) which focused on resilience and building capacity for women in food security among others (USAID, 2018). The RISE‐REGIS‐Accelerated Growth (RISE‐REGIS‐AG) program focused on strengthened resilience to environmental, security and economic shocks for 19,355 stakeholders in the same three regions from 2015 to 2020.

In Tahoua, Maradi and Zinder there is an ongoing program (2019–2023) on Building Community Resilience in Niger. The project covers 900,000 people in target areas and aims to strengthen resilience and systems at the community, municipality, district, regional, and national levels. Activities include supporting integrated health and nutrition services for mothers and children (e.g., IYCF, breastfeeding, nutrition counselling, micronutrient supplementation), education, and WASH.

In addition to the multiregional programs mentioned above, Maradi was the target for The Livelihoods, Agriculture and Health Interventions in Action (LAHIA) program which focused on malnutrition prevention among pregnant and lactating mothers and children under two in from 2012 to 2017. It was designed to reach 17,972 children (6–23 months old) and 20,076 pregnant and lactating mothers with a health, nutrition, hygiene, and sanitation package complemented by supplemental food rations and a protective household ration provided during the lean season (USAID, Catholic Relief Services, 2017).

In Zinder from 2013 to 2017, Initiative d'accélération des Objectifs du millénaire pour le développe‐ment au Niger (IAOMD) was implemented and reported reaching 801,000 children and 844,000 women of reproductive age high chronic malnutrition. Interventions consisted of community mobilization and behaviour change activities in the areas of maternal and infant nutrition, nutrition education, family practices, gardening, and WASH. The project distributed micronutrient supplementation and improved antenatal and post‐natal care services at the community level.

Programs also targeted social norms and gender roles that influence behaviour and practices related to nutrition, feeding (breastfeeding and complementary feeding), and caregiving. For instance, women and young children are traditionally allocated smaller portions of quality food compared to men, especially in large and polygamous households. One such program was Programme d'Appui à la Sécurité Alimentaire des Ménages Tanadin Abincin Iyali (PASAM‐TAI) implemented by Catholic Relief Services from 2012 to 2018. Objectives included reducing chronic malnutrition in households with pregnant and lactating women and children under 5 years, increasing production and consumption of food for nutrition and income, improving disaster risk management, and expanding gender roles for women and men to enhance sustainable results (USAID, Catholic Relief Services, 2017). The program successfully piloted a curriculum called Strengthening Marriages and Relationships through Planning and Communication (SMART) for which evidence showed improvements in participating couples' communication and shared decision‐making abilities for household and health‐related decisions (USAID, Catholic Relief Services, 2017).

### 3.3 Maternal and neonatal nutrition outcomes

#### 3.3.1 Food security

In 2017, the most recent nationwide estimates show approximately a third of the population was classified as at risk of food insecurity, 12% moderately food insecure and only 3% severely food insecure (CC/SAP/PC, Republique du Niger, INS‐Niger, 2017). Despite declines in moderate food insecure households since 2010 (30% in 2010 to 12% in 2017), there remains evidence of widespread insecurity throughout the last decade (CC/SAP/PC, Republique du Niger, INS‐Niger, 2017). Time trend data are not available for household dietary diversity, but in 2015, nearly 80% of rural households had low or medium level diet diversity (32% low: 47% medium) (Institut National de la Statistique INS & Ministere de la Sante Publique, 2020).

**Figure 2:**
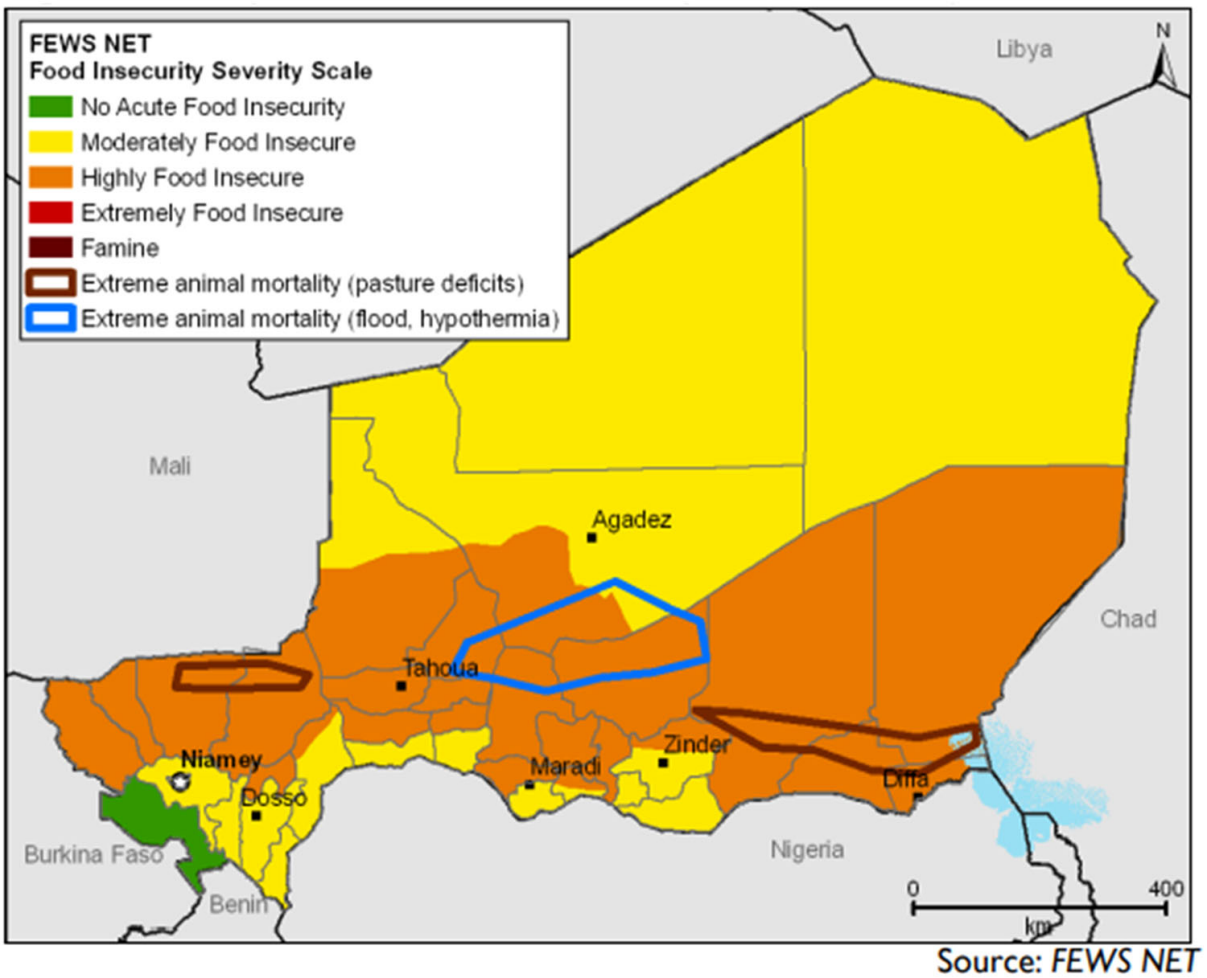
FEWS NET food insecurity severity scale 2011.

Regions of Tillaberi, Tahoua, Maradi, and Zinder were more impacted by food insecurity during the 2005 and 2010 food crisis with Integrated (Acute Food Security) Phase Classification (IPC) Phase 3 levels^1^ and above (Figure 2). FEWS NET food security maps from late 2010 classify most of the country as either moderate or highly food insecure. The southern regions were hardest hit along with large pockets of extreme animal mortality from pasture deficits in Zinder, Diffa and Tillaberi and animal mortality from floods in Tahoua and Maradi. Recognized as the most vulnerable regions, they were subsequently targeted for interventions by government and development partners

With the prolonged and repeated crises in Niger, acute events lead to chronic challenges. An official from the General Directorate of Population and Reproductive Health exhibited concerns about the frequency of climate shocks and the impact on nutrition:

“At the beginning, [droughts] were every 5 years, but now it's more and more every 2 to 3 years…. The [wasting] threshold set by the WHO is 20% for us to talk about a nutritional crisis, but we are already at 45%, so Niger is really the only country of its kind.”

#### 3.3.2 National maternal nutrition trends

Iron folate supplementation during pregnancy for 90+ days doubled from 14% in 2006 to 29% in 2012 and then dropped in 2021 to 21%; anaemia during pregnancy remains high and essentially unchanged (62% in 2006, 59% in 2012, and 57% in 2020). Overall, the proportion of underweight women (BMI < 18.5) decreased from 14% in 2006 to 10% in 2012 and prevalence of short stature (<145 cm in height) in Niger is very low (<1% of women of reproductive age, 2012). The proportion of adolescent girls aged 15–17 with BMI‐for‐age <−2 SD according to WHO 2007 child growth standards has remained the same during the period under consideration: 6% in 2006 and 7% in 2012 (DHSs).

**Figure 3:**
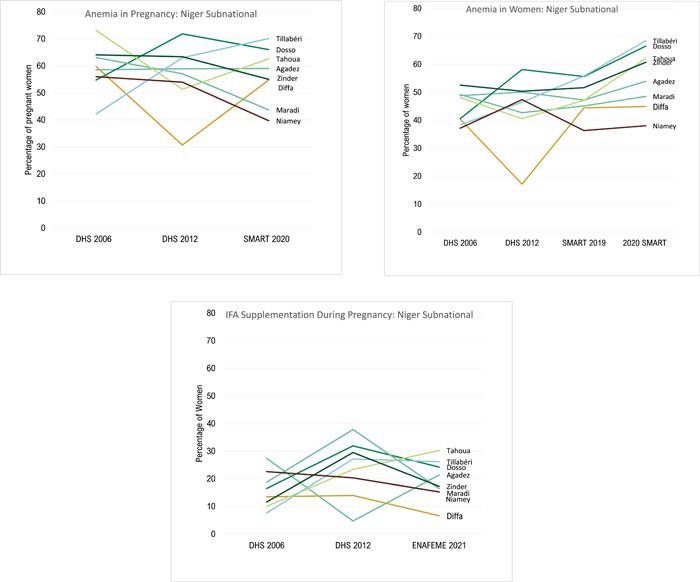
Maternal nutrition subnational trends from 2006 to 2021 on anaemia and IFA supplementation.

The latest 2020 SMART survey shows that nationally only 53% of women achieve minimum dietary diversity for women (MDD‐W) (ranging from 28% in Maradi to 83% in Diffa). This suggests that diet quality, including achieving adequate micronutrient intake, is a problem in Niger with large differences among regions. Dietary diversity data are available for 2020 only (Figure 3).

#### 3.3.3 Subnational maternal nutrition trends

There were large decreases in anaemia during pregnancy in Maradi (63% in 2006 to 44% in 2020), Niamey (56% in 2006 to 40% in 2020), Tahoua (73% in 2006 to 63% in 2020), and Zinder (64% in 2006 to 40% in 2020). Coverage of iron supplementation from 2006 to 2012; in Tahoua coverage increased by 13% points (pp) (from 10% in 2006 to 23% in 2012), in Maradi coverage increased by 19pp (from 19% in 2006 to 38% in 2012) and Zinder increased by 18pp (from 12% in 2006 to 30% in 2012). Subnational gains of coverage of iron supplementation from 2006 to 2012 were not sustained into 2021 in most of the regions. Anaemia in all women (aged 15‐49) is however a different story with no gains from 2006 to 2020, in fact, there are a greater percentage of women with anaemia in 2020 (Figure 3). Underweight in women (27% in 2006 to 8% in 2012) reduced in Diffa (Supporting Information: Figure 1).

#### 3.3.4 National neonatal nutrition trends

There were marked improvements in breastfeeding practices since around 2000. Early initiation of breastfeeding has increased from 47% in 2006 to 74% in 2018 but then declined slightly to 68% in 2021. The percentage of children born in the previous 2 years who received something other than breast milk in the first 3 days of life decreased from 80% in 2006 to 49% in 2012. Exclusive breastfeeding has increased from around 14% in 2006 to 24% in 2021 for babies less than 1 month of age.

#### 3.3.5 Subnational neonatal nutrition trends

**Figure 4:**
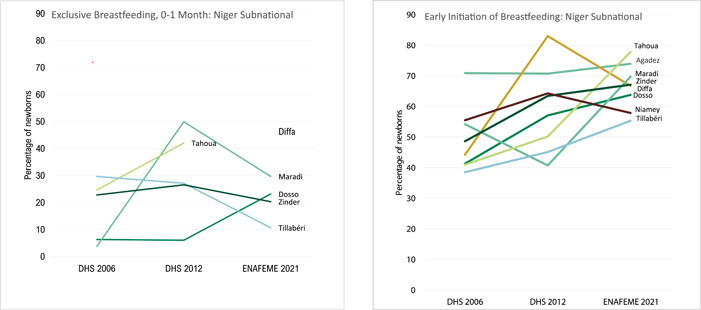
Neonatal nutrition subnational trends from 2006 to 2021.

With early initiation of breastfeeding, there were large improvements in Tillabéri (39%% in 2006 to 55% in 2021), Diffa (44% in 2006 to 67% in 2021), Dosso (41% in 2006 to 64% in 2021), Maradi (54% to 2006 to 70% in 2021), Tahoua (41% in 2006 to 78% in 2021), and Zinder (49% in 2006 to 67% in 2021) (Figure 4). There were improvements in exclusive breastfeeding for 0–1 months in Maradi (4% in 2006 to 50% in 2012 to 30% in 2021) and Tahoua (25% in 2006 to 42% in 2012) although data from other regions are not available due to limited sample size from the surveys. Available subregional and national breastfeeding indicators suggest gains in exclusive breastfeeding for 0–1‐month‐olds.

#### 3.3.6 Subnational trends in newborn growth faltering in stunting and wasting

In Niger, there is a slight birth disadvantage in child HAZ (i.e., children being born stunted; mean HAZ = ~0.5) in all regions which remains consistent over time suggesting that maternal health and nutrition may not have improved notably across the years (Supporting Information: Figure 2). Note that data was not available for Zinder region.

**Figure 5:**
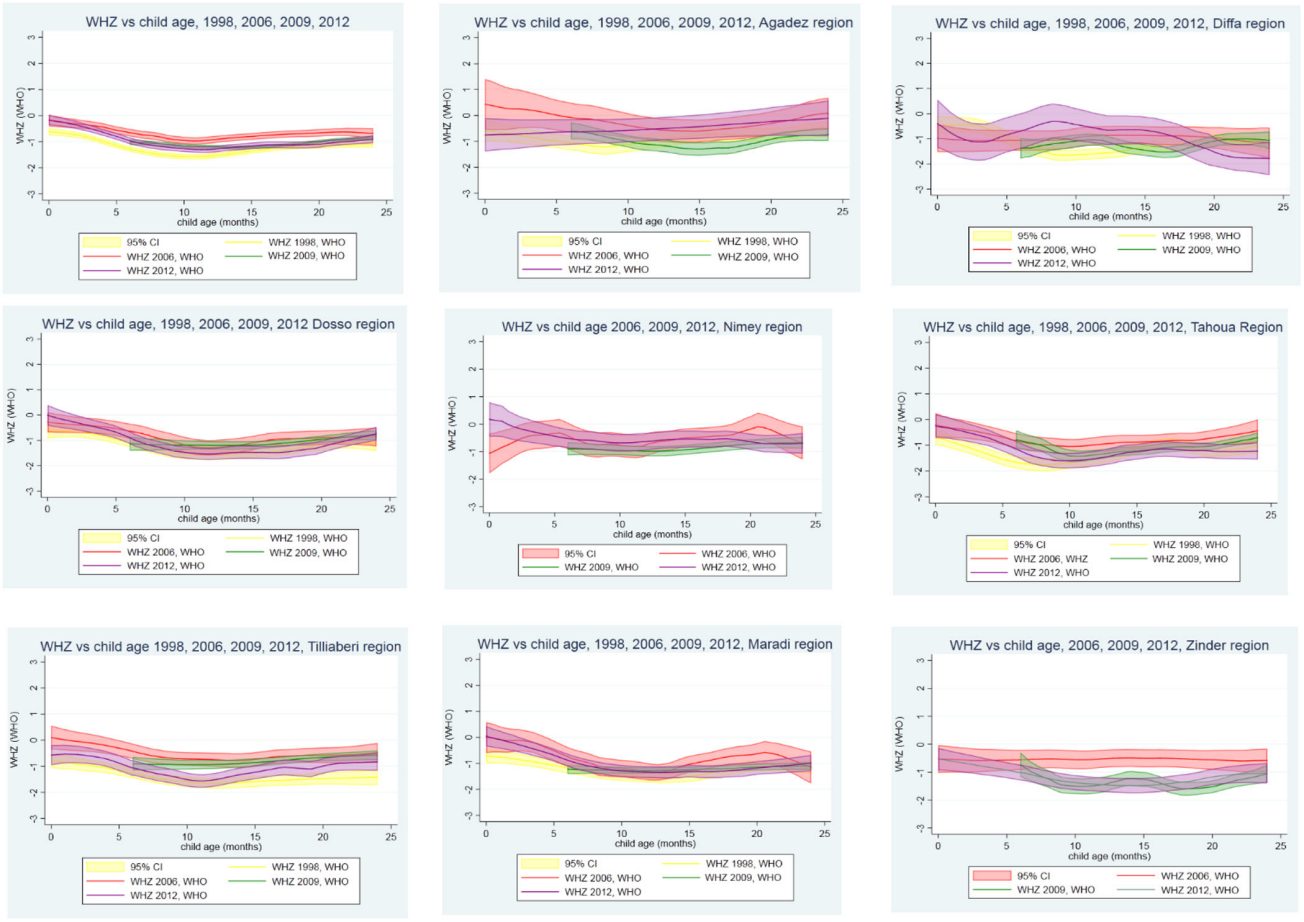
Child weight for height *z*‐score (WHZ) growth faltering trends in Niger 1998, 2006, 2009 and 2012.

For wasting (WHZ) in Niger, all regions have a severe birth disadvantage (mean WHZ = ~−0.6) in 1998 suggesting that children were being born wasted (Figure 5). At the national level, the birth disadvantage improved significantly from −0.6 (95% confidence interval [CI]: −0.5, −0.8) to −0.2 (95% CI: 0, −0.4) in 2012, but varied by region. By 2012, the birth disadvantage disappeared completely in Dosso and Maradi regions and improved significantly in Tahuoa region (Figure 5). Maternal health and nutrition interventions in those regions may have played a strong role in improving fetal growth. Agadez, Diffa and Tillaberi regions saw no change in child wasting at birth by 2012.

## 4 DISCUSSION

First, we do not have data to assess the complete knowledge of the implementation successes, challenges and outcomes of all the nutrition and food security programs. Therefore, it is difficult to ascertain attribution to improvements in nutrition and food security and resilience. However, the findings suggest a significant contribution to these improvements given the multiplicity of programs, the diversity of actors, the extent of financing, and the targeting of vulnerable populations in a complex environment. This also reinforces the need to explore subnational trends when seeking to understand nutritional influences on changes in maternal and newborn mortality.

Second, our analysis applied a novel approach to understand in‐utero fetal growth and neonatal growth as there is limited data on maternal weight gain weight gain during pregnancy, in‐utero fetal growth or size at birth/birthweight in Niger and across low‐income‐countries. One way to better understand some of these prenatal phenomena is to examine child anthropometry versus age curves. For the newborn, this can shed light on the birth disadvantage, that is, to what extent the newborn is deficient in weight or growth compared to optimal health newborns of globally recognized standards.

Third, before 2006, Niger has limited data to track nutritional maternal and newborn indicators. Therefore, national and subnational trends and differences in maternal and newborn nutritional status were drawn for the period of 2006–2021. In response to the chronic climate crisis, the Nigerien government and program implementers have demonstrated their commitment to reducing food insecurity and enhancing resilience to climate shocks by adopting a deliberate multisectoral policy and program effort. The investments in maternal health and nutrition interventions in targeted regions (i.e., Maradi, Tahou, Tillaberi and Zinder) may have played a meaningful role in improving food security, maternal nutrition, fetal growth and neonatal health and likely helped offset any substantial rise in nutrition related maternal and newborn mortality caused by the repeated food crises in the hardest hit regions (Table 1).

The idea of food security has been central in the discussion on sustainable development. The Sustainable Development Goal (SDG) 2 for ‘Zero Hunger’ aims to ‘End hunger, achieve food security and improved nutrition and promote sustainable agriculture’ (United Nations, 2015). Although some progress has been made over the last decades, food security is still a major issue with as many as 811 million people in the world facing hunger in 2020 (Republic du Niger, DNPGCA, 2020). Nearly one in three people in the world (2.37 billion) did not have access to adequate food in 2020—an increase of almost 320 million people in just 1 year. Indeed, climate change has a role to play in agriculture and food security through natural resource availability and agricultural production (Steffen et al., 2015; United Nations, 2015). Without appropriate attention, financing, and effective intervention coverage, climate change will continue to affect food security and nutrition. Major drivers of food insecurity and malnutrition, such as conflict, climate variability and extreme economic hardships (currently worsened by the COVID‐19 pandemic) are only expected to increase in the coming years and countries will need to prepare for future shocks.

Evidence shows that climate shocks influence nutritional status, which reinforces the importance of strong investments in disaster risk reductions to build resilience and respond to disasters to mitigate negative impacts (Fanzo & Downs, 2021). Over the last 10 years, Niger has committed to several proactive measures such as improving early warning systems, standardizing, coordinating, and monitoring food security data, and strengthening disaster preparedness.

The Intergovernmental Panel on Climate Change (IPCC) highlights that undernutrition due to climate shocks may be one of the most significant consequences due to the vast number of people who may be impacted (Smith et al., 2014). In Niger, children aged 2 or younger born in a drought were 72% more likely to be stunted (Watkins, 2007). This reinforces the importance of food security and nutrition during pregnancy. In Niger, our results showed some improvements in underweight and anaemia during pregnancy. Although we did not explore child stunting rates in our research as our focus was on the mother and newborn, we did assess trends in children being born stunted and found similar associations with climate shocks and nutritional status. In other high climate vulnerable countries, Ethiopia and Kenya, there is evidence that children born during a drought are 36%–50% more likely to be malnourished compared to their nondrought born counterparts (Smith et al., 2014). Since 2020, Niger has also endured the shock of the COVID‐19 pandemic with major economic, food, and health systems disruptions impacting food security and nutrition outcomes. The National Food Crisis Prevention and Management System (Republic du Niger, 2020) in Niger predicts that an additional 2.6 million people may face a food crisis during the lean period (an increase from 2 million to 5.6 million people).

Although Niger has made strong investments, the current situation is still devastating with reports of the worst food security crisis in a decade, with a projected 3.6–4.4 million food insecure people, according to the IPC findings. This is largely due to the chronic cycle of delayed and irregular rainy seasons and drought. Escalating security threats have led to instability and lack of access in the Tillaberi, Tahoua, Diffa and Agadez regions due to the continuous deterioration since 2012. The humanitarian crisis has also led to population displacement with 580,838 people of concern with 48% internally displaced, 43% refugees, 6% returnees and 2% asylum seekers (UNHCR, 2022). While natural disasters cannot currently be controlled, programs have worked to strengthen households' and communities' ability to prepare for and withstand shocks by prioritizing disaster risk reduction, strengthening early warning systems, and building local capacity and resilience.

While the focus of this paper and analysis was on maternal and newborn nutrition and food security, it is critical to consider how maternal and newborn health and nutrition links to child and adolescent nutrition. While wasting persists throughout Niger, there has been some progress in reducing child wasting from 16.2% in 2000 to 9.8% in 2019 (Global Nutrition Report, 2022). The predominantly pastoralist regions of Zinder, Maradi and Tahoua continue to be the hardest hit and challenges persist with coverage of wasting services. Yet, the early adoption and application of community management of acute malnutrition continue and the government's commitment to ensure service availability has contributed to the mortality reduction in children under five (Concern Worldwide, Irish Aid, 2021).

## 5 CONCLUSION

Despite focused and deliberate policies and programs to strengthen food security in Niger since 2000, variations in maternal and neonatal nutrition indicators (and consequent maternal and neonatal mortality rates) suggest that these are acute, suboptimal and short‐lived. Repetitive and chronic crises, escalating security threats and cultural barriers, among others, continue to hinder progress. While pathways from maternal and newborn nutrition to survive and thrive are clear, only a deliberately focused approach will bend the maternal and neonatal mortality curve in a high‐risk environment such as Niger. There should be a continued and sustainable focus on vulnerable regions to build resilience, targeting populations at high risk, and investments in infrastructure to improve health systems, food systems, agriculture systems, education systems, and social protection.

## AUTHOR CONTRIBUTIONS

Shelley Walton, Nasreen S. Jessani, Heather Jue‐Wong, Nadia Akseer, Elizabeth A. Hazel and Agbessi Amouzou designed the study. Shelley Walton, Nasreen S. Jessani, Heather Jue‐Wong, Elizabeth A. Hazel, Assanatou Bamogo, and Nadia Akseer conducted analyses. Shelley Walton, Nasreen S. Jessani, Heather Jue‐Wong, Nadia Akseer wrote the first draft of the manuscript. And all authors edited and revised the draft manuscript and approved the final manuscript.

## CONFLICT OF INTEREST STATEMENT

The authors declare no conflict of interest.

## Supporting information

Supporting information.

Supporting information.

Supporting information.

## Data Availability

The data that support the findings of this study are openly available in The DHS Program at https://dhsprogram.com/data/available-datasets.cfm.

## Footnotes

1 Integrated (Acute Food Security) Phase Classification (IPC): Food Security Modern prediction and classification of risk of famine is guided by the IPC system which aggregates, synthesizes and provides a basis to interpret data on food consumption patterns, coping behaviours, prevalence rates of acute undernutrition (wasting malnutrition), destitution and mortality. An IPC level 3 is facing critical levels of food insecurity.
