# Supplementary material for: Climate shocks and nutrition: The role of food security policies and programs in enhancing maternal and neonatal survival in Niger
Source: Matern Child Nutr. 2023 Oct 4;20(1):e13566. doi: 10.1111/mcn.13566 (PMC10750024; doi:10.1111/mcn.13566)
Supplement: Supplementary file 1 — Supporting information. [file MCN-20-e13566-s003.docx]

**Supplemental Figure 1.** Subnational trends from 2006 to 2012 on underweight women
