# Supplementary figures and images for: Climate shocks and nutrition: The role of food security policies and programs in enhancing maternal and neonatal survival in Niger
Source: Matern Child Nutr. 2023 Oct 4;20(1):e13566. doi: 10.1111/mcn.13566 (PMC10750024; doi:10.1111/mcn.13566)

**Supplemental Figure 2**. Child growth faltering trends in Niger – height for age z-score (HAZ), (1998,2006, 2009 2012)


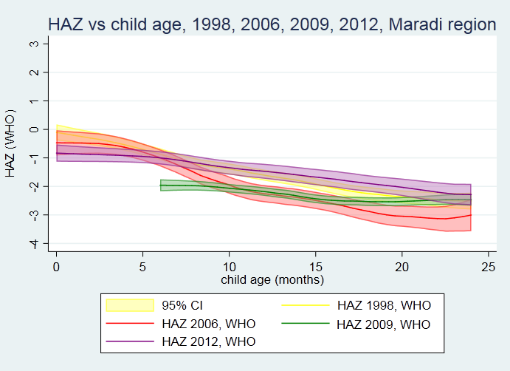

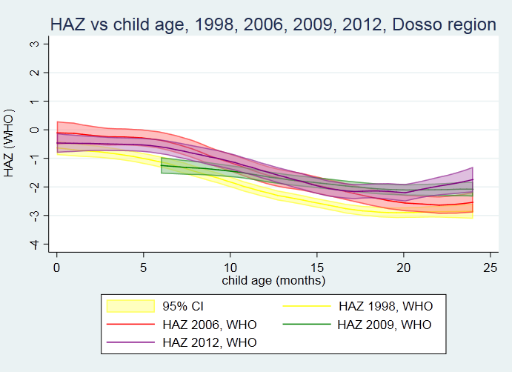


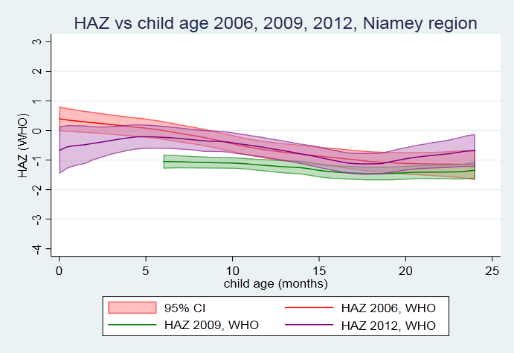

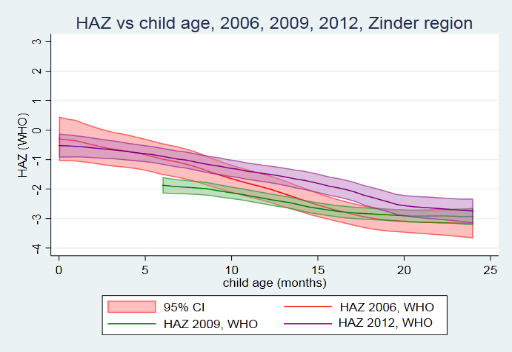

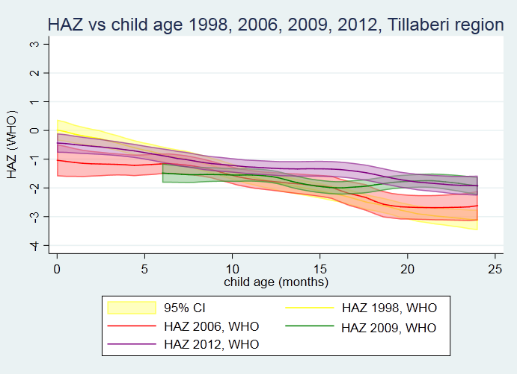

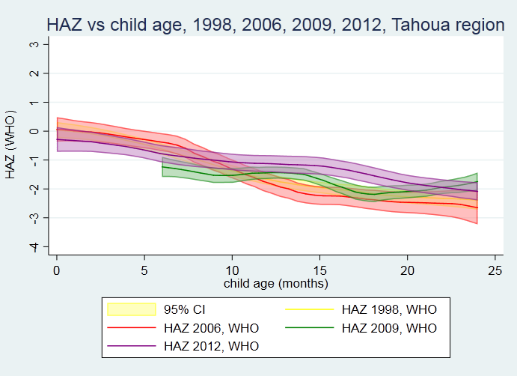


***
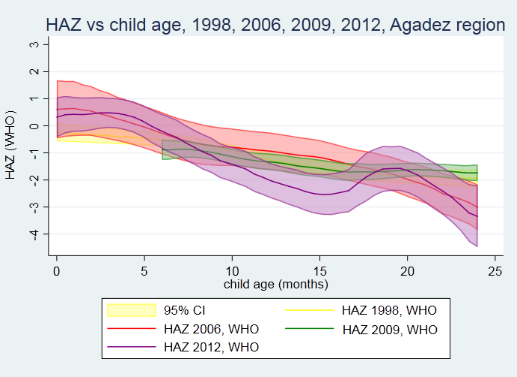
***
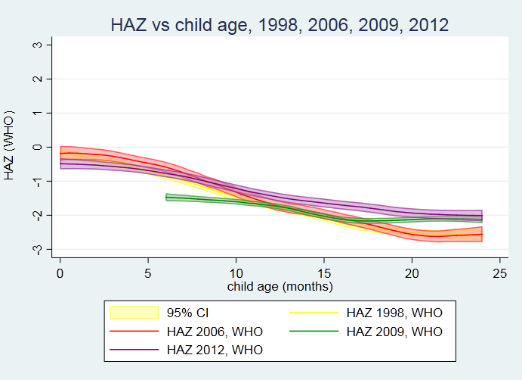

Supplement: Supplementary file 2 — Supporting information. [file MCN-20-e13566-s001.docx]
