# Supplementary material for: Climate shocks and nutrition: The role of food security policies and programs in enhancing maternal and neonatal survival in Niger
Source: Matern Child Nutr. 2023 Oct 4;20(1):e13566. doi: 10.1111/mcn.13566 (PMC10750024; doi:10.1111/mcn.13566)
Supplement: Supplementary file 3 — Supporting information. [file MCN-20-e13566-s002.docx]

**Supplemental Table 1**. Maternal, newborn, and child nutrition, food security, and resilience programs in Niger

| **Program** | **Years** | **Program partners (funding and implementing organizations)** | **Interventions targeted maternal health, neonatal health, and/or resilience** | **Location** | **Reported impact** |
| --- | --- | --- | --- | --- | --- |
| Ongoing work of World Food Program (WFP) in Niger | 1968-present | WFP | MH, NH, Resilience | National | WFP’s investments in nutrition in Niger have aimed to provide immediate aid in emergencies and strengthen resilience long term. WFP nutrition programs focus on children 6-23 months, pregnant and lactating women, and adolescent girls and align with Niger’s National Security Nutrition Policy (WFP, 2016).  In 2015, WFP began emergency response operations in the Diffa region providing food aid to refugees and internally displaced people affected by Boko Haram attacks (WFP, 2017). The regional operation provided unconditional and conditional food and cash distributions, nutritional supplementation for children under 2 years of age, and school meals.  WFP’s Country Strategic Plan for Niger (2020-2024) outlines priorities for achieving the following results: everyone has access to food, no one suffers from malnutrition, food systems are sustainable, countries strengthened capacities, and global partnership (WFP, 2019).  In 2021, WFP assisted 75,000 people with unconditional food and cash distributions during the pastoral lean season and 240,000 during the agricultural lean season, and 500,000 through the Food Assistance for Assets program (WFP, 2021). WFP supported trainings on malnutrition screening and referral with the Food Crises Unit/National System for the Prevention and Management of Food Crises and Nutrition Directorate. |
| Ongoing work of Food and Agriculture Organization of the United Nations (FAO) in Niger | 1978-present | FAO | MH, Resilience | National | FAO has primarily focused on issues of food and nutrition security in Niger. One targeted project has improved access to fertilizers, seeds, and financial and advisory services for 100,000 smallholder farmers in agricultural villages in 2008-2013 (FAO, 2015). Farmers were trained in new agricultural techniques and business management skills. FAO also introduced micro-finance schemes for eligible farmers and aimed to increase and diversify agricultural, livestock, forestry, and fishery production. FAO has developed national policies with the government and other partners for nutrition and social protection. |
| Livelihoods, Agriculture and Health Interventions in Action (LAHIA) | 2012-2017 | USAID Office of Food for Peace, Save the Children, World Vision | MH, NH | Maradi | LAHIA’s goal was to reduce food insecurity and malnutrition by targeting poor and rural households. The project focused on chronic malnutrition among pregnant and lactating mothers and children below the age of five years, specially focusing on children below two years. The project also increased availability and access by diversifying food production and increasing targeted households’ incomes. LAHIA established Women’s Savings and Loans Groups to support women in businesses and Husbands’ School to involve men in decisions related to reproductive health and promote shared household decision-making. It was designed to reach 17,972 children (6-23 months old) and 20,076 pregnant and lactating mothers with a health, nutrition, hygiene and sanitation package complemented by supplemental food rations and a protective household ration provided during the lean season (USAID & SAVE THE CHILDREN, 2017). |
| Programme d’Appui à la Sécurité Alimentaire des Ménages-Tanadin Abincin Iyali (PASAM-TAI) | 2012-2017 | USAID Office of Food for Peace, Catholic Relief Services | MH, NH | Maradi, Zinder | PASAM-TAI aimed to address food security and malnutrition by improving WASH, mainly promoting handwashing and eliminating open defecation. WASH and nutrition activities targeted pregnant mothers and children under five years of age. The project also aimed to improve nutrition education and behaviors and increase food production in communities. As a result, 71 villages were certified open defecation free, 15 wells were rehabilitated, 3 mini-piping of potable water were constructed, and 24 school latrines were built (USAID & Catholic Relief Services 2017). |
| Initiative d’accéléra-tion des Objectifs du millénaire pour le développe-ment au Niger (IAOMD) | 2013-2017 | European Union, UNICEF | MH, NH | Zinder | IAOMD focused on delivering essential nutrition interventions and reported reaching 801,000 children and 844,000 women of reproductive age in municipalities with high chronic malnutrition. Interventions consisted of community mobilization and behavior change activities in the areas of maternal and infant nutrition, nutrition education, family practices, gardening, and WASH (USAID & SPRING, 2018). The project distributed micronutrient supplementation and improved antenatal and postnatal care services at the community level. |
| Resilience and Economic Growth in the Sahel – Enhanced Resilience (RISE-ER) | 2013-2018 | USAID, National Cooperative Business Association Cooperative League of the United States | Resilience | Tillaberi, Maradi, Zinder | The first RISE Initiative, RISE-ER, was a flagship multisectoral resilience project designed to reduce chronic vulnerability by improving economic well-being, strengthening institutions and governance, and improving health and nutrition status in 25 targeted commune communities. The project focused on strengthening households and villages’ capacity to recover from shocks and stresses – instead of being set back by each repeated emergency – by addressing root causes of chronic vulnerability and supporting sustainable livelihoods (NCBA CLUSA, 2021). |
| Capacity Building for Community Prevention and Management of Crises and Disasters in Niger | 2014 | Oxfam, Karkara | Resilience | National | Advocacy activities involved supporting the decentralization of the National Mechanism for the Prevention and Management of Food Crises and Disasters and encouraged local level development planning. Advocacy results were 23 vulnerability monitoring observatories, 46 community early warning and emergency response systems operational in 23 municipalities, and over 100 NGO, government, and technical partners trained (Oxfam, 2016). Capacity building activities included training of stakeholders and resulted in 3 partner organizations supported in community monitoring mechanisms of vulnerability and emergency response. Overall program results consisted of strengthened linkages between local and national structures to monitor vulnerability and the integration of a disaster risk reduction approach in local development plans by municipalities. |
| RISE Initiative: Sahel Resilience Learning Project (SAREL) | 2014-2019 | USAID, The Mitchell Group | Resilience | Tillaberi, Maradi, Zinder | RISE Initiative: SAREL strengthened the capacity of key stakeholders through trainings in evidence-based methods and innovations for governance and building resilience. The project was an effort to integrate humanitarian and development assistance, promote local ownership, and address gender issues important to achieving resilience in agricultural and agro-pastoral livelihood zones (USAID, 2018). |
| RISE Initiative: Resilience in the Sahel Enhanced – Accelerated Growth (REGIS-AG) | 2015-2020 | USAID, Cultivating New Frontiers in Agriculture | Resilience | Tillaberi, Maradi, Zinder | RISE Initiative: REGIS-AG targeted chronically vulnerable areas in efforts to increase household incomes in agricultural and agro-pastoral areas vulnerable to crisis. The project aimed to increase household incomes by improving cowpea, poultry, and small ruminant value chains and exports (CNFA, 2018). |
| Building Community Resilience in Niger | 2019-2023 | German Cooperation, UNICEF | MH, NH, Resilience | Maradi, Zinder, Tahousa | The project covers 900,000 people in target areas and aims to strengthen resilience and systems at the community, municipality, district, regional, and national levels (German Cooperation, 2020). Activities include supporting integrated health and nutrition services for mothers and children (e.g., IYCF, breastfeeding, nutrition counseling, micronutrient supplementation), education, and WASH. The project builds capacity and accountability mechanisms with local authorities along with multi-sectoral capacity at the regional and national levels, to prepare for and respond to future shocks and crises. |

References

CNFA (2018). Resilience and Economic Growth in the Sahel - Accelerated Growth. Retrieved from <https://www.cnfa.org/program/resilience-and-economic-growth-in-the-sahel-accelerated-growth/>

Food and Agriculture Organization of the United Nations. (2015). Niger and FAO, partnering for sustainable development and food and nutrition security. Retrieved from <http://www.fao.org/3/BC031E/bc031e.pdf>

German Cooperation, République du Niger, UNICEF. (2020). Building Community Resilience in Niger. Retrieved from <https://www.unicef.org/niger/media/5161/file/Building%20resilience%20in%20Niger.pdf>

NCBA CLUSA. (2021). Resilience and Economic Growth in the Sahel - Enhanced Resilience (REGIS-ER) Final Report. Retrieved from <https://ncbaclusa.coop/content/uploads/2021/04/REGIS-ER-Final-Report-April-2021.pdf>

Oxfam. (2016). Capacity Building for Community Prevention and Management of Crises and Disasters in Niger. Oxfam Novib Case Study. Retrieved from <https://policy-practice.oxfam.org/resources/capacity-building-for-community-prevention-and-management-of-crises-and-disaste-594569/>

USAID & Catholic Relief Services. (2017). Programme d’Appui à la Sécurité Alimentaire des Ménages-Tanadin Abincin Iyali (PASAM-TAI). Retrieved from <https://pdf.usaid.gov/pdf_docs/PA00TBBZ.pdf>

USAID & Save the Children. (2017). LAHIA Non-Emergency Food Assistance Program. Quarterly Report - FY17 Q1 (October-December 2017. Retrieved from <https://pdf.usaid.gov/pdf_docs/PA00MPC3.pdf>

USAID. (2018). Sahel Resilience Learning. Final Performance Evaluation. Retrieved from <https://www.globalwaters.org/sites/default/files/Sahel-Resilience-Learning-Final-Performance-Evaluation.pdf>

USAID & SPRING. (2018). Drivers of Malnutrition in Niger: Analysis of Secondary Data Sources. Retrieved from

<https://www.spring-nutrition.org/sites/default/files/publications/briefs/spring_drivers_of_malnutrition_in_niger_0.pdf>

World Food Programme. (2016). Nutrition in Niger, strengthening resilience by investing in nutrition. Retrieved from <https://docs.wfp.org/api/documents/WFP-0000019583/download/>

World Food Programme. (2017). WFP Response in Diffa Factsheet. Retrieved from

<https://www.wfp.org/publications/2017-niger-fact-sheets>

World Food Programme. (2019). Niger Country Strategic Plan (2020-2024). Retrieved from

<https://www.wfp.org/operations/ne02-niger-country-strategic-plan-2020-2024>

World Food Programme. (2021). WFP Niger Country Brief June 2021. Retrieved from

<https://docs.wfp.org/api/documents/WFP-0000130477/download/>
